# Environmental change and the rate of phenotypic plasticity

**DOI:** 10.1111/gcb.16291

**Published:** 2022-06-21

**Authors:** Tim Burton, Irja Ida Ratikainen, Sigurd Einum

**Affiliations:** ^1^ Centre for Biodiversity Dynamics, Department of Biology Norwegian University of Science and Technology Trondheim Norway; ^2^ Norwegian Institute for Nature Research Trondheim Norway

**Keywords:** acclimation, acclimation rate, acclimation time‐course, phenotypic plasticity, plasticity rate, rapid environmental change, timescale of plasticity

## Abstract

With rapid and less predictable environmental change emerging as the ‘new norm’, understanding how individuals tolerate environmental stress via plastic, often reversible changes to the phenotype (i.e., reversible phenotypic plasticity, RPP), remains a key issue in ecology. Here, we examine the potential for better understanding how organisms overcome environmental challenges within their own lifetimes by scrutinizing a somewhat overlooked aspect of RPP, namely the rate at which it can occur. Although recent advances in the field provide indication of the aspects of environmental change where RPP rates may be of particular ecological relevance, we observe that current theoretical models do not consider the evolutionary potential of the rate of RPP. Whilst recent theory underscores the importance of environmental predictability in determining the slope of the evolved reaction norm for a given trait (i.e., how much plasticity can occur), a hitherto neglected possibility is that the rate of plasticity might be a more dynamic component of this relationship than previously assumed. If the rate of plasticity itself can evolve, as empirical evidence foreshadows, rates of plasticity may have the potential to alter the level predictability in the environment as perceived by the organism and thus influence the slope of the evolved reaction norm. However, optimality in the rate of phenotypic plasticity, its evolutionary dynamics in different environments and influence of constraints imposed by associated costs remain unexplored and may represent fruitful avenues of exploration in future theoretical and empirical treatments of the topic. We conclude by reviewing published studies of RPP rates, providing suggestions for improving the measurement of RPP rates, both in terms of experimental design and in the statistical quantification of this component of plasticity.

## Graphical abstract

Individuals respond to environmental change via phenotypic plasticity, the magnitude of which is measured as the slope of the relationship describing phenotypic expression across environments (a). Yet, plasticity also takes time to manifest and the rate at which it occurs (b) might be as critical in enabling organisms to cope in new environments. However, we understand little about plasticity rates in an ecological or evolutionary context. By discussing why plasticity rates might evolve and providing guidelines for standard and comparable measurements, we hope to stimulate theoretical development and testing of novel hypotheses about organismal performance under environmental change.

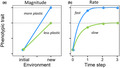

## 1 INTRODUCTION

Individuals experiencing deteriorating local environments can respond in the short term via two primary routes: ‘escape’, by relocating elsewhere, or ‘tolerance’ of local environmental stress. For many organisms, the possibility for escape is limited or not an option, and they must often rely on tolerating environmental change by reversibly altering their physiological, behavioural and/or morphological phenotype (Gabriel, 2005; Gabriel et al., 2005; Padilla & Adolph, 1996; Siljestam & Östman, 2017). Initial theory proposed that such reversible phenotypic plasticity (hereafter, RPP) may yield substantial benefits when environmental fluctuations are large, predictable and occur slowly relative to the rate at which the phenotype can be changed, because it should allow the phenotype to track the optimum that changes in concert with the environment (Gabriel, 2005; Gabriel et al., 2005; Padilla & Adolph, 1996; Siljestam & Östman, 2017). With the increasing magnitude and pace of global environmental change as a backdrop, understanding how organisms respond to environmental change within their own lifetimes has gained immense impetus among biologists in recent years (Diamond & Martin, 2021; Fox et al., 2019; Pinek et al., 2020; Snell‐Rood et al., 2018). To date, substantial emphasis has been placed on explaining variation, both among species and within species, in the capacity for RPP (i.e., the degree to which a phenotypic trait can be changed, mostly commonly envisaged as the reaction norm slope). This has seemingly occurred because verbal arguments have been used to morph the ‘latitudinal variability’ hypothesis (Janzen, 1967) with more quantitative models of RPP (e.g., Gabriel, 2005, 2006; Padilla & Adolph, 1996) into the prediction that species or populations inhabiting more variable environments should have evolved a greater capacity for RPP (Box 1). However, despite rigorous experimental investigation, support for this prediction remains weak (Gunderson & Stillman, 2015; Kelly et al., 2012; MacLean et al., 2019; Pereira et al., 2017; Phillips et al., 2016; Sgro et al., 2010; van Heerwaarden et al., 2014; van Heerwaarden et al., 2016). Yet, this focus on capacity for RPP has perhaps overshadowed consideration of the *rate* at which organisms can respond plastically to environmental change. In other words, it is inherently assumed that in a fluctuating and periodically stressful environment, organisms will always have sufficient time to mount the phenotypic changes, be they large or small, required for persistence. We propose that this is an assumption that warrants further exploration, and that depends on the speed with which organisms can actually change their phenotypes.

BOX 1 **Reversible phenotypic plasticity and environmental variability**

In 1967, Daniel Janzen developed an influential, largely verbal hypothesis in his article ‘Why Mountain Passes Are Higher in the Tropics’ (Janzen, 1967). Janzen's hypothesis draws upon the notion that topographical barriers to individual dispersal likely depend on the magnitude of the temperature gradient across them rather than the actual change in altitude. Furthermore, based upon the observation that annual variation in ambient temperature tends to be low in tropical versus temperate locations, Janzen proposed that there is less overlap in temperature between lowland and upland areas in the tropics. As a part of his hypothesis and of more relevance to the issue at hand, Janzen offered the prediction that organisms in the tropics should thus evolve narrower thermal tolerances and reduced acclimation capacities (i.e., lower capacity for RPP), a phenotype likely complementary to the less variable climate of low‐latitude regions (Janzen, 1967). Although intuitive, Janzen's model does not consider the degree of predictability associated with variation in the environment, something early quantitative modelling subsequently suggested to be a critical factor in understanding the evolution of RPP (or acclimation as referred to by Janzen). Thus, RPP in a given trait was proposed to be beneficial in variable environments that fluctuate predictably and at a slow rate relative to the speed with which the phenotype can actually be adjusted (Gabriel, 2005, 2006; Padilla & Adolph, 1996; Siljestam & Östman, 2017). However, such models are rarely acknowledged in empirical tests of the ‘latitudinal variability hypothesis’. In the vast majority of cases, no distinction is made between the level of variability versus predictability in the environmental parameter of interest, either conceptually or in terms of experimental design (for an exception, see Phillips et al., 2016). Nor is the actual rate of phenotypic adjustment relative to the timescale of environmental change considered.

## 2 THE RATE OF PLASTICITY IN AN ECOLOGICAL SETTING

Reversible plasticity is often quantified using reaction norms, where the phenotypic value of a given trait (e.g., metabolic rate) is expressed as a function of an environmental variable (e.g., temperature). Reaction norms have been implemented as a tool across biological disciplines to better understand and forecast the performance, distribution and extinction risk of species (Huey et al., 2012; Schou et al., 2017; Valladares et al., 2014). Reaction norms tend to be measured in stable conditions, whereas natural environments are the opposite—they tend to fluctuate. Unsurprisingly, there are several instances where reaction norms have been shown to be relatively poor predictors of ecological phenomena in more natural (i.e., fluctuating) environments (Ketola & Kristensen, 2017; Ketola & Saarinen, 2015; Kingsolver et al., 2015; Sinclair et al., 2016). Possible reasons for such discrepancies have been discussed in detail elsewhere (Ketola & Kristensen, 2017; Sinclair et al., 2016). However, for the case at hand, one reason stands out—for reaction norms to predict organismal performance in fluctuating environments, RPP must either be instantaneous or at least as fast as the rate of environmental change. Yet, this seems unlikely to apply universally. For example, following a change in its environment, an individual must first collect and process information about its new environment. Then the individual needs to initiate a response that might involve changes in gene expression, neural function and production of hormones/enzymes/proteins that are required to yield the necessary shifts in whole organism phenotype. Intuitively, it seems plausible that the performance or fitness consequences may arise when an individual's phenotype ‘lags’ behind the optimum for the current environment and recent evidence seems to indicate this. For example, Kronholm and Ketola (2018) demonstrated that growth under variable temperature in the fungus *Neurospora crassa* depends on the frequency of temperature fluctuations relative to the rate at which growth can be adjusted. Thus, reductions in growth were observed when the timescale of temperature change was less than the rate at which growth could be adjusted. Similar evidence comes from a study of experimental communities composed of different cyanobacteria species. Each species varied in the rate at which it could adjust its photosynthetic pigmentation in response to light coloration. Under exposure to different frequencies of change between red and green coloured light, the most phenotypically flexible of these species was able to competitively exclude the others when the timescale of change in light coloration exceeded that required for the flexible species to completely adjust its pigmentation (Stomp et al., 2008). Yet, quantitative theory that considers this rate of phenotypic response in a broader ecological context is scarce. Accordingly, there has been scant consideration of the types of environments in which rates of RPP may be of particular importance. However, several recent contributions have developed verbal predictions describing the types of environments where rates of RPP may be of ecological significance (Fey et al., 2021; Kremer et al., 2018; Pinek et al., 2020). In two of these key papers, these predictions were tested by measuring the growth rate of experimental populations of phytoplankton that differed in initial phenotype (as induced by prior acclimation to different temperatures) and were exposed to different patterns of temperature fluctuation (e.g., temperature could fluctuate in a particular direction or the amplitude or frequency of temperature cycles could vary). Observed values of population growth were then compared with predictions generated by mechanistic models that either considered or ignored the rate of plasticity in population growth, which was estimated indirectly from the experimental data (Fey et al., 2021; Kremer et al., 2018). The environmental domains where the rate of plasticity in population growth held ecological importance were then assumed to be indicated where models that accounted for the rate of plasticity were able to outperform models that either disregarded the rate of plasticity or assumed that it occurred instantaneously. This process of confronting predictions with experimental data indicated that the ecological consequences associated with rates of plasticity likely depend on interactions between the past environments experienced by individuals in a population (because this will influence the ‘initial’ phenotype of those individuals and thus determine how much phenotypic change will be required to produce the new phenotype in the new environment) and the magnitude, frequency and direction of change in the current environment (Fey et al., 2021; Kremer et al., 2018).

## 3 CAN THE RATE OF PLASTICITY EVOLVE?

Despite the fundamental advances offered by the work of Kremer et al. (2018), Pinek et al. (2020) and Fey et al. (2021), the reality remains that ecological processes can be influenced by evolution (and vice versa, Govaert et al., 2019). This raises the enticing possibility that future investigation into the ecological implications associated with rates of RPP may benefit from the development of quantitative theory that also considers the evolutionary potential of this component of plasticity. For example, early models recognized that the fitness payoff resulting from RPP is likely dependent on the rate at which the phenotype can be adjusted (e.g., Gabriel, 2006; Gabriel et al., 2005). Yet, current theory assumes that rates of RPP are adaptively inert with respect to variation in the timescale of environmental change (Siljestam & Östman, 2017). However, if one considers the breadth of known evolutionary innovation, it seems premature to dismiss the possibility that rates of RPP might evolve in response to the pace of environmental change and thus aid organisms in adjusting their ability to tolerate environmental stress. Preliminary empirical evidence supports this possibility. Different strains of the fungus *Neurospora crassa* vary in the rate at which they can adjust growth in response to temperature change (Kronholm & Ketola, 2018), different strains of the bacterium *Staphylococcus aureus* show variation in the rate of growth plasticity in response to antibiotic treatment (Yang et al., 2021) and populations of the broadly distributed estuarine fish *Fundulus heteroclitus* reveal distinct variation in the rate at which they can perform physiological adjustments in response to osmotic stress (Whitehead et al., 2012).

Here, we provide a brief overview of some recent theoretical models that consider the evolution of phenotypic plasticity, giving a concise summary of each model and its key results, as they relate to rates of plasticity. Lande (2009), Ezard et al. (2014) and Tufto (2015) use quantitative genetic approaches to model the evolution of irreversible phenotypic plasticity, where the phenotype is determined by the environment experienced during development, and selection on the phenotype occurs during a later stage. Such models implicitly assume that sufficient time elapses between the time of plasticity induction and the time at which selection occurs and are as such not directly relevant for the topic discussed here. However, it is worth noting that an important determinant of the evolutionary outcome for such models is the predictability of the environmental change over the time lag between these two events. Phenotypic plasticity, in terms of the slope of the evolved reaction norm, increases in strength with increasing predictability in the environment (Lande, 2009). We propose that these models already hint at the ecological importance of the rate of plasticity: organisms that can rapidly implement their phenotypic response to an environmental cue may initiate this process closer to the time of selection than organisms with a slower rate of phenotypic response. In effect, this should make it simpler for the faster responding organism to more accurately ‘predict’ the future selective environment (assuming sufficient environmental autocorrelation exists) which may, in turn, influence the shape of the evolved reaction norm.

The possible importance of predictability in the evolution of plasticity rates is further underscored by Botero et al. (2015), who applied a simulation modelling approach to investigate how the evolution of plasticity depends not only on the predictability of the environment, but also the rate of environmental change relative to generation time. Populations that initially consisted of genotypes with different reaction norm intercepts and slopes, as well as different abilities to exhibit reversible plasticity, were allowed to evolve (in the presence of mutational input and plasticity costs) under different environmental scenarios. These analyses show that reversible plasticity will only evolve when environmental predictability is over a certain threshold, and then only if environments change rapidly relative to the generation time such that an individual experiences repeated shifts in environments throughout life. Above a certain level of predictability, evolution resulted in transitions from reversible to irreversible plasticity and eventually flat reaction norms with decreasing rates of environmental change. As with the previously discussed models, Botero et al. (2015) did not consider the rate of plasticity explicitly. Rather, the model simulations were conducted in a stepwise manner, where genotypes that expressed plasticity completely adjusted their phenotype according to the environmental cue experienced in the previous time step and their genotypic reaction norm. Thus, assuming there is genetic variance in the rate of plasticity, it remains unclear how the rate of plasticity might evolve and contribute to the evolution of reaction norms. Again however, it can be argued that environmental predictability may be intrinsically linked to the rate of plasticity; a high rate of plasticity in this case means that two successive time steps are effectively ‘closer together in time’, which may actually serve to increase environmental predictability.

A clear conclusion from these models is that environmental predictability is of high importance for understanding the evolution of plasticity. However, a less intuitive but perhaps equally important insight is that environmental predictability in the context of phenotypic plasticity is not only a characteristic of the environment, but also a combined outcome of autocorrelation in the environment *and* the rate of plasticity. Thus, in future theoretical treatments it might be possible to consider environmental predictability as a more dynamic parameter of the model. Indeed, the importance of the rate of plasticity for environmental predictability was made explicit in the quantitative genetics model of Lande (2014), which considers traits that undergo continuous reversible plasticity and selection. Environmental predictability in that model, averaged over development time, increases with the rate of plasticity. Mirroring the models above, it predicts (assuming a cost of plasticity) that higher environmental predictability leads to a steeper reaction norm. Thus, this model illustrates the importance of the rate of plasticity for understanding the evolution of reaction norms. It does not, however, address optimality of this rate, nor its evolutionary dynamics in different types of environments. Lastly, a notable omission from quantitative models which describe the conditions under which RPP is expected to evolve is the role of behaviour, despite its known potential to dampen the effects of the environment on other components of the phenotype (Buckley et al., 2015; Enriquez‐Urzelai et al., 2020; Fey et al., 2019; Muñoz et al., 2016; Sears et al., 2016). While a detailed consideration of this issue is beyond the scope of the current article, considerable potential likely exists for behaviour to influence the evolution of RPP (in terms of both its capacity and now rate, as proposed here) in other traits. Thus, there is clearly great scope for further insight by developing quantitative models and empirical studies which consider scenarios where both the rate and capacity for plasticity can coevolve and where individual behaviour has the potential to influence the dynamics of this relationship.

## 4 RAPID REVERSIBLE PHENOTYPIC PLASTICITY: IS IT COSTLY?

Reversible plasticity is an effective means of buffering environmental change, and hence reducing environmental variance in population growth rate. Yet, its rate and magnitude presumably have limits, otherwise genetic evolution would be redundant as a response to environmental change. Evolutionary theory suggests that this limitation may arise through a trade‐off between environmental variance in population growth rate vs. intrinsic population growth rate and carrying capacity in the average environment (Lande, 2009). Accordingly, reducing the impact of environmental variation through phenotypic plasticity can only be achieved by simultaneously reducing fitness in a stable environment. This introduces the concept of the cost of plasticity as a main driver behind the evolution of plasticity, which can be categorized into costs of maintenance and costs of production. For maintenance costs, more plastic genotypes must invest more resources in maintaining the ‘machinery’ needed to detect, monitor and respond to environmental conditions. This cost will be paid in all environments, whereas less plastic genotypes are not encumbered with such an investment (Auld et al., 2010). On the other hand, production costs are those paid by a given genotype when adjusting its phenotype. Given that production costs are only paid when the plastic response is triggered, and which are then usually more than outweighed by the fitness benefits provided by the plasticity, maintenance costs shape the evolution of plasticity to a much greater extent than production costs (Sultan & Spencer, 2002). We suggest that this maybe a crucial point in better understanding the evolution of the capacity for phenotypic plasticity versus the rate at which it occurs.

Assuming that the capacity for plasticity can be increased most simply by operating the ‘machinery’ required to change that trait for longer, then it will mostly be the costs of production that increase. In contrast, to boost the rate of change in the same trait necessitates increasing the size or output of that ‘machinery’, which will likely be associated with costs even when the machinery is inactive. Thus, we propose that the proportional increase in maintenance cost maybe larger for increasing the rate than for increasing the capacity for plasticity. If this is correct, this may be crucial for understanding the evolution of plasticity in general, as well as how we should approach our study of it. Specifically, populations living in stable environments may pay a relatively small price for maintaining their capacity to produce plastic phenotypic change, and adaptation of this trait to level of environmental fluctuations may be relatively weak. This possibility is supported by a meta‐analysis which shows a weak or absent relationship between the capacity for plasticity and fitness in stable environments (Van Buskirk & Steiner, 2009). In contrast, populations living in more stable environments should experience strong selection against maintaining rapid plasticity due to high maintenance costs. Adaptative evolution across populations may then be expected to be more pronounced for the rate of plasticity than for the capacity. Given that the potential for the rate of RPP to evolve is absent from current theory, we urge renewed theoretical and empirical considerations of this topic so that the possibilities put forward here can be evaluated more formally.

## 5 MEASURING THE RATE OF REVERSIBLE PLASTICITY

**FIGURE 1:**
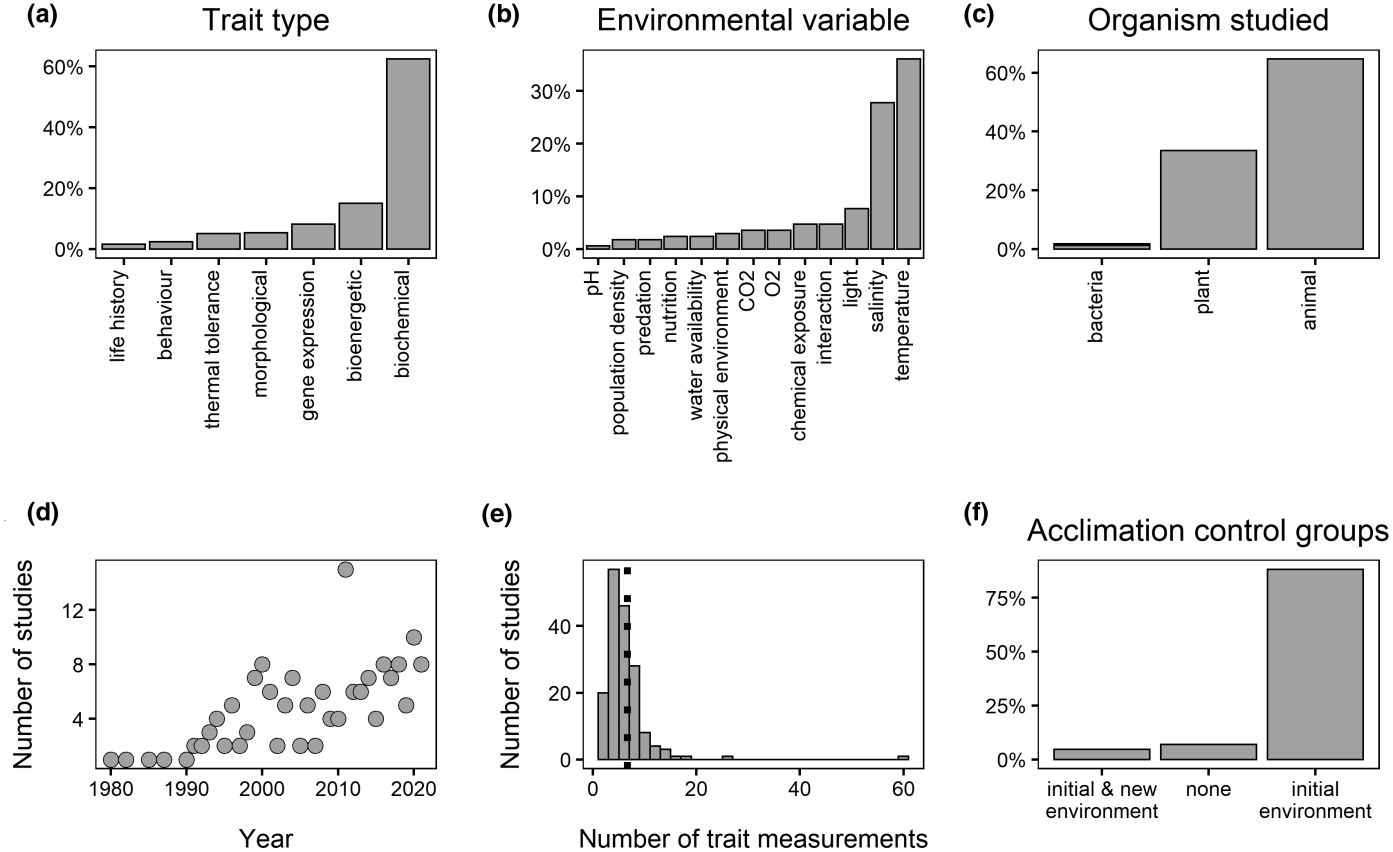
(a) Proportion of all phenotypic traits measured when grouped by trait category. A total of 578 trait measurements were reported in the 170 studies reviewed. (b) Proportion of studies manipulating one of the 13 categories of environmental variables described in the supplementary text. Only one study, Becker et al. (2011) performed separate manipulations of two environmental variables and is excluded from this plot. (c) Proportion of studies (*n* = 170) implementing either bacteria, plant or animal species as the study organism. (d) Scatterplot showing number of empirical studies investigating the rates of phenotypic plasticity published per year since 1980. (e) Histogram showing the mean number of measurements of the phenotype made following the shift from the initial to the new environment (i.e., during the time course of acclimation). A mean value is presented for each of the 170 studies because in some cases data were reported on multiple phenotypic traits within the same study, of which the number of measurements performed during the time course of acclimation could vary from trait to trait. The dashed line indicates the overall mean. (f) Proportion of studies implementing either no control group, a control group acclimated to the ‘initial’ environment or two control groups, one acclimated to the ‘initial’ environmental state and one acclimated to the ‘new’ environmental state. A full description of this literature search is contained in the supplementary material. Data are available in the dryad digital repository (Burton et al., 2022).

Interest in empirical measurements of the timescale of RPP dates back by at least a century (e.g., Loeb & Wasteneys, 1912). However, to our knowledge, even a basic synthesis of this literature is absent. We surveyed 170 empirical studies that investigated RPP rates (a full description of this literature search is contained within the supplementary text), observing that (i) biochemical traits tend to be the most frequently measured class of trait (Figure 1a), (ii) temperature and salinity have been the most commonly studied environmental variables (Figure 1b), (iii) animals have been employed more often than plants or bacteria as the study organism of choice (Figure 1c) and (iv) since the 1980s, there has been a slight increase in the number of studies investigating rates of plasticity each year (Figure 1d). Remarkably, only five of the studies identified in our literature survey (i.e., 2.9%) present formal statistical quantification of the rate of plasticity in the trait under examination (Layne & Claussen, 1987; Londos & Brooks, 1990; Pintor et al., 2016; Sandblom et al., 2014; Yang et al., 2021). This deficiency in quantifying the rate of RPP suggests that we may require a better understanding of the shape of plastic responses shown when phenotypes acclimated to an initial environment approach a new phenotype in the new environment.

**FIGURE 2:**
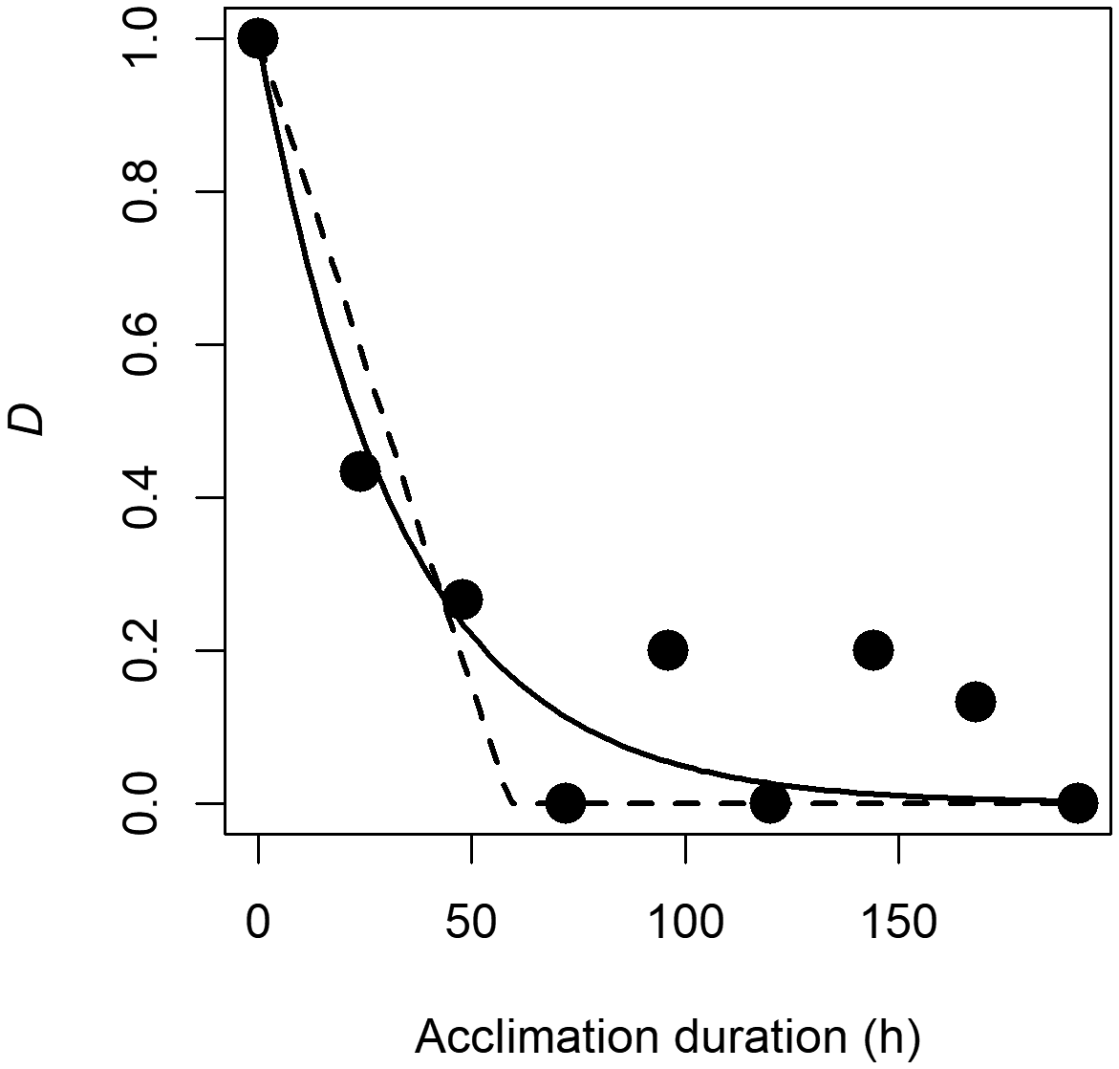
Illustration of the proposed method for quantifying the rate of phenotypic plasticity based on the temporal change in values of D, which gives the proportion of the full plastic response that remains to be achieved after varying durations of exposure to a new environment following prior acclimation to an initial environment (see main text for calculation). Data are from one of the experiments presented by Kuyucu and Chown (2021), where the insect species *Mucrosomia caeca* was first kept at 10 °C (‘initial environment’) before being shifted to 20°C (‘new environment’). The minimum critical temperature was then determined for individuals after different durations of acclimation to 20°C. Solid and dashed lines represent fitted exponential decay and segmented regressions, respectively. Here, the exponential decay function yielded the best fit (residual standard errors: Exponential 0.106, segmented regression 0.123), and gave an estimated λ of 0.03021 h^−1^. This corresponds to a half‐time of 22.9 h (i.e., the time taken for the deviation from the phenotype acclimated to the initial environment, to be reduced by 50% following the shift to the new environment, given as ln(2)/λ)).

Lande (2014) assumed that at any given time following a shift in the environment, the rate of change in the phenotype due to plasticity is proportional to the difference between the current phenotype and the fully developed plastic phenotype. In this case, the rate of phenotypic change can be modelled as an exponential decay function, dD_t_/dt = − λD_t_, where λ is the rate of plasticity, and D_t_ is the proportion of the full plastic response that remains to be achieved at time t. Thus, D_t_ is given by (z_t_‐ z_∞_)/(z_0_ – z_∞_), where z_0_ is the first measurement of the phenotype in the new environment, z_t_ is the phenotype at an intermediate time point t and z_∞_ is the fully adjusted phenotype in the new environment. Note that z_0_ does not need to be measured at any particular timepoint following transfer into the new environment, since the exponential decay rate is assumed to be constant. Thus, for a given observed value of D_t_ after time t, the rate of plasticity can be calculated as λ = −ln(D_t_)/t. In cases where plasticity appears to proceed more linearly towards the new phenotype (e.g., De Meester & Cousyn, 1997), the plasticity rate is given by λ = (1‐ D_t_)/t. However, from an organismal (fitness) perspective, exponential decay and linear rates of phenotypic change are not equivalent, because the initial approach towards the fully adjusted phenotype is more rapid under exponential decay, and thus the two rates cannot be directly compared. Furthermore, care should be taken to not assume the wrong shape of plastic response when estimating such rates: in most cases, the shape of the plastic response will not be known a priori, in which case phenotypes should be measured at multiple values of t. For such datasets, modelling using nonlinear regression (for exponential decline) and segmented regression (for linear decline) can be applied to determine the shape of plasticity. Since D_t_ per definition is fixed at 1 for t = 0, both these models will estimate a single parameter, and their relative fit can be compared directly using the residual standard errors (see Figure 2 for an example of how this procedure can be applied to experimental data). In relation to this point, our literature search revealed that of the 170 studies surveyed, phenotypes were on average, measured 6.6 times following manipulation of the environment (Figure 1e), suggesting that determining the shape of plasticity can be done with existing data. Furthermore, the suggested approach for comparing shapes and quantifying rates of plasticity is only applicable to traits that experience a unidirectional change in trait values following a shift in the environment. Although this is perhaps the most common type of response within the realm of ecology (e.g., change in morphology, behaviour or higher order physiological traits such as thermal tolerance), many biochemical responses to environmental shifts involve active up/downregulation or passive distortion of molecule and ion concentrations followed by a return towards initial values via homeostatic regulation. For such responses, it is perhaps more difficult to envision a standardized measure of RPP rates that can be compared across studies and species.

Based on the above, we outline some suggestions for future experimental work on rates of RPP. First, for estimation of the rate of plasticity to be comparable across studies, it should be expressed on a proportional scale relative to the maximum plasticity possible in the new environment. This requires measurements of phenotypes that have had sufficient time to acclimate to the new environment (z_∞_). Most previous studies lack this information, which greatly reduces their comparability. In our literature survey, we observed that it is common to implement only a single control group when measuring rates of plasticity (i.e., a group acclimated to the ‘initial environment’ experienced by the study organisms, Figure 1f). This means that the study organisms are acclimated to the initial environmental state for a given period of time before a subsample is transferred to the ‘new’ environmental state, after which a time course of phenotypic measurements is made on both groups. While this type of design reveals phenotypic change in relation to the initial environment, it does not provide information on the magnitude of phenotypic change achievable in the *new* environment (and thus does not allow estimation of the true rate at which the ‘new’ phenotype can be approached). Very few studies implement the second group of control individuals that is necessary to obtain such information (i.e., groups acclimated to both the ‘initial and new’ environments experienced, Figure 1f). Based on the above considerations, we provide three recommendations for future studies of rates of plasticity. First, phenotypic measurements should be made on samples of study organisms that have also been a priori acclimated to the ‘new’ environment. Second, measurements of the phenotype in the new environment should be performed on several occasions during the process of acclimation to obtain information on the shape of the plastic response (i.e., exponential decay vs. linear), and ideally provide fits of statistical models that can distinguish between these alternatives. And third, once the shape of the phenotypic response is determined, the corresponding procedure outlined above should be used to estimate λ, as such estimates will provide a quantitative basis for future comparative analyses of the rate of RPP.

## 6 CONCLUSION

Given that phenotypic plasticity may be instrumental in helping organisms ‘buy time’ when accruing evolutionary adaptations to novel environments (e.g., Diamond & Martin, 2021), a substantial gap in our understanding of how plasticity may actually facilitate this clearly exists. However, recent advances have shed light on the types of ecological conditions where the rate of the phenotypic plastic response is likely of importance. Thus, by re‐evaluating current theory so that it better considers the possibility that the timescale of RPP might evolve (along with consideration of possible costs involved) and implementing a more systematic approach in our measurement of plasticity rates, we may be able to test novel, more ecologically relevant predictions regarding the capacity of organisms to tolerate the unprecedented rates of environmental change that are currently occurring in natural environments. By addressing these knowledge gaps and methodological considerations, we will likely improve our understanding of how organisms tolerate environmental change in their own lifetimes, which may ultimately contribute to informing evidence‐based policies aimed at mitigating the effects of anthropogenically induced environmental change on natural populations and ecosystems.

## Supporting information


Appendix S2

Appendix S2

Appendix S2

Appendix S2

## Data Availability

The data that support the findings of this study are available at https://doi.org/10.5061/dryad.tdz08kq2d.
