# Supplementary material for: Environmental change and the rate of phenotypic plasticity
Source: Glob Chang Biol. 2022 Jun 21;28(18):5337–45. doi: 10.1111/gcb.16291 (PMC9541213; doi:10.1111/gcb.16291)
Supplement: Supplementary file 2 — Appendix S2 [file GCB-28-5337-s002.docx]

Supplementary methods, gives full details of the literature search and data extraction procedure described in the main text

Supplementary data extracted from papers identified in literature search according to the criteria described in the supplementary methods

Supplementary references – papers from which supplementary data were extracted using procedure described in supplementary methods
