# Supplementary material for: Environmental change and the rate of phenotypic plasticity
Source: Glob Chang Biol. 2022 Jun 21;28(18):5337–45. doi: 10.1111/gcb.16291 (PMC9541213; doi:10.1111/gcb.16291)
Supplement: Supplementary file 3 — Appendix S2 [file GCB-28-5337-s004.docx]

Supplementary material: Environmental change and the rate of phenotypic plasticity

Tim Burton, Irja Ida Ratikainen and Sigurd Einum

*Measuring the rate of reversible plasticity*

The data summarized in Figure 1 in the main text was obtained from a literature search performed in December 2021. To identify published empirical research focusing on the rate of phenotypic plasticity, we entered the search terms in Web of Science: (ALL= ("acclimation" or "phenotypic plasticity") AND ALL = ("time-course*" or "time-scale*" or "time-period*" or "response-time*")). We opted to exclude the word “rate” from this search due to the high number of false results that were returned in preliminary trials. This initial search yielded 885 papers. We then scanned the title and abstract (if available) of these papers, excluding cases where it could be determined unambiguously that the rate of plasticity was not a focus of the study. This reduced the initial list to 276 papers. Due to time constraints, we excluded studies where the full text version of the paper could not be located online (n = 16) and then scanned the text of each of the remaining papers, omitting those that (i) were review articles, (ii) implemented a gradual (rather than acute) change in the environmental variable under manipulation, (iii) were performed in field conditions, (iv) did not contain data showing a time-course of change in the phenotypic variable(s) of interest and (v) implemented only a transient treatment exposure to the environmental variable of interest (e.g. heat shock experiments). This yielded the final list of 170 studies. We note here that we are aware of several studies, predominantly published prior to 1980 (Brattstrom & Lawrence, 1962; Brett, 1946; Cossins, Friedlander, & Prosser, 1977; Hutchison, 1961; Layne, Manis, & Claussen, 1985), that investigate rates of plasticity but which were not identified by these search criteria.

From each of the 170 papers, we then extracted data for the variables listed below. The abbreviated name used in the database accompanying this paper is stated in parentheses, along with a brief description of the values each variable could assume: year of publication (***year***: integer), taxonomic group *(****taxon.group***: categorical - plant/animal/bacteria), species name (***species***: character), environmental variable subject to manipulation (***env.var***: character), phenotypic trait(s) measured (***trait***: character), the acclimation status of the control groups (***acc.type.control***: categorical - initial-environment/initial- and new-environment/none), number of phenotypic measurements made during time-course of acclimation to new environment (***timepoints.treatment***: integer), statistical quantification of the rate of plasticity (***rate.quant***: categorical - yes/no), reference (***ref***: character*)*.

Exploration of the extracted data revealed a high diversity of phenotypic traits. To aid quantitative interpretation, each of the recorded traits was placed into one of 7 categories. Where a given grouping maybe ambiguous, we list several illustrative examples below. The categories were: life history (e.g. body size, population growth rate), behaviour (e.g. prey search time, swimming speed), thermal tolerance (e.g. CTmin, heat knockdown time), morphological (e.g. body shape, leaf thickness), gene expression (e.g. mRNA level associated with a given gene), bioenergetic (e.g. traits involved in photosynthesis, respiration or energy conversion) and biochemical (e.g. enzyme activities, fluid osmolality, fluid ion concentration). Likewise, a high diversity of environmental variables was also evident in the extracted data. We thus categorized these variables into 13 groups. Again, where there may be potential for ambiguity in a given grouping, we list several examples. The 13 groups were: *pH*, *carbon dioxide concentration*, *oxygen concentration*, *population density*, *water availability, temperature*, *predation* (e.g. predator kairomones), *nutrition* (e.g. food quantity, food quality or nitrogen availability), *chemical exposure* (e.g. exposure to copper or aluminum ions), *light* (e.g. light intensity, level of UV radiation) and *salinity* (i.e. salt concentration). Two final groups that require further explanation are *physical environment* (e.g. water flow, background color) which includes aspects of the physical environment not covered by any of the previous groupings, and a group we termed *interaction* to describe instances where two environmental variables were subject to simultaneous experimental manipulation (e.g. temperature × food availability). This variable is termed ***env.var.category*** (character variable). Data is available in the Dryad digital repository (Burton, Ratikainen, & Einum, 2022).

**References**

Brattstrom, B. H., & Lawrence, P. (1962). The rate of thermal acclimation in anuran amphibians. *Physiological Zoology, 35*(2), 148-156. doi:10.1086/physzool.35.2.30152723

Brett, J. R. (1946). Rate of gain of heat-tolerance in goldfish (*Carassius auratus*). *Publications of the Ontario Fisheries Research Laboratory, 64*, 5-28.

Burton, T., Ratikainen, I. I., & Einum, S. (2022). *Data from: Environmental change and the rate of phenotypic plasticity*. doi:<https://doi.org/10.5061/dryad.tdz08kq2d>

Cossins, A. R., Friedlander, M. J., & Prosser, C. L. (1977). Correlations between behavioral temperature adaptations of goldfish and the viscosity and fatty acid composition of their synaptic membranes. *Journal of comparative physiology, 120*(2), 109-121. doi:10.1007/BF00619309

Hutchison, V. H. (1961). Critical thermal maxima in salamanders. *Physiological Zoology, 34*(2), 92-125.

Layne, J. R., Manis, M. L., & Claussen, D. L. (1985). Seasonal variation in the time course of thermal acclimation in the crayfish *Orconectes rusticus*. *Freshwater Invertebrate Biology, 4*(2), 98-104. doi:10.2307/1467181
